# RNA-Binding Proteins as Novel Effectors in Osteoblasts and Osteoclasts: A Systems Biology Approach to Dissect the Transcriptional Landscape

**DOI:** 10.3390/ijms251910417

**Published:** 2024-09-27

**Authors:** Anastasia Meshcheryakova, Serhii Bohdan, Philip Zimmermann, Markus Jaritz, Peter Pietschmann, Diana Mechtcheriakova

**Affiliations:** 1 Department of Pathophysiology and Allergy Research, Center of Pathophysiology, Infectiology and Immunology, Medical University of Vienna, 1090 Vienna, Austriapeter.pietschmann@meduniwien.ac.at (P.P.); 2 Immunai/Nebion AG, 8048 Zürich, Switzerland; phz@nebion.com; 3 Research Institute of Molecular Pathology, Vienna Biocenter, 1030 Vienna, Austria; markus.jaritz@imp.ac.at

**Keywords:** RNA-binding proteins, osteoclasts, osteoblasts, bone homeostasis, bone remodeling, transcriptional profile, transcriptomics, systems biology

## Abstract

Bone health is ensured by the coordinated action of two types of cells—the osteoblasts that build up bone structure and the osteoclasts that resorb the bone. The loss of balance in their action results in pathological conditions such as osteoporosis. Central to this study is a class of RNA-binding proteins (RBPs) that regulates the biogenesis of miRNAs. In turn, miRNAs represent a critical level of regulation of gene expression and thus control multiple cellular and biological processes. The impact of miRNAs on the pathobiology of various multifactorial diseases, including osteoporosis, has been demonstrated. However, the role of RBPs in bone remodeling is yet to be elucidated. The aim of this study is to dissect the transcriptional landscape of genes encoding the compendium of 180 RBPs in bone cells. We developed and applied a multi-modular integrative analysis algorithm. The core methodology is gene expression analysis using the GENEVESTIGATOR platform, which is a database and analysis tool for manually curated and publicly available transcriptomic data sets, and gene network reconstruction using the Ingenuity Pathway Analysis platform. In this work, comparative insights into gene expression patterns of RBPs in osteoblasts and osteoclasts were obtained, resulting in the identification of 24 differentially expressed genes. Furthermore, the regulation patterns upon different treatment conditions revealed 20 genes as being significantly up- or down-regulated. Next, novel gene–gene associations were dissected and gene networks were reconstructed. Additively, a set of osteoblast- and osteoclast-specific gene signatures were identified. The consolidation of data and information gained from each individual analytical module allowed nominating novel promising candidate genes encoding RBPs in osteoblasts and osteoclasts and will significantly enhance the understanding of potential regulatory mechanisms directing intracellular processes in the course of (patho)physiological bone turnover.

## 1. Introduction

The strength and quality of the bones making up the skeleton is ensured by continuous bone remodeling processes, which represent the central part of the bone homeostasis. To preserve the bone structure, the osteoclast-driven bone resorption is paralleled by the osteoblast-mediated bone formation. The activity of osteoclasts and osteoblasts is regulated by osteocytes and their products. Furthermore, as highlighted in the field of osteoimmunology, components of the immune system also have significant impact on bone [1,2,3,4]. These tightly regulated bi-directional processes are interconnected by so-called coupling factors. Prominent in this respect is the receptor activator of NF-κB (RANK)/receptor activator of NF-κB ligand (RANKL)/osteopotegerin (OPG) axis [5,6]. Emerging discoveries in the field of bone research further highlight sphingosine-1-phosphate (S1P), one of the central bioactive lipid mediators of the cellular sphingolipid system, as a promising candidate interconnecting the counteracting players osteoclasts and osteoblasts [7]. Aberrations in the process of cell differentiation, in the cell count, in the recruitment, and in the activity of bone-resolving and bone-forming cells may lead to pathophysiological conditions such as osteoporosis. Osteoporosis-associated bone fragility results in a high risk of bone fractures in the affected patients [8,9]. Treatment options include anabolic drugs that support bone formation and anti-catabolic medication that is intended to decrease bone resorption. Additional treatment strategies make use of monoclonal antibodies targeting sclerostin, a product of osteocytes [10,11,12].

MicroRNAs (miRNAs) represent an important level of the regulation of gene expression. These small non-coding RNAs are complementary to the target sequence in the mRNA and act by repressing translation and/or triggering the degradation of the corresponding mRNA. Central to this is the RNA-induced silencing complex, RISC [13,14]. The miRNA-driven knockdown of target genes is a critical event in multiple cellular and biological processes [15]. Thus, miRNAs have been highlighted as crucial regulators in several severe multifactorial diseases such as cancer [16,17,18], autoimmunity [19,20], and, importantly for this study, osteoporosis [21,22,23].

The biogenesis of miRNAs is regulated by RNA-binding proteins (RBPs). Treiber T et al. [24] applied a proteomics-based pull-down approach and identified a compendium of 180 RBPs that interact with miRNA precursors; those proteins were found to show specific binding to individual miRNA precursors or to a subset of miRNA precursors and might either fulfill the housekeeping functions or be involved in the cell type-, tissue type- or disease-specific regulatory mechanisms [24]. The role of this set of RBPs in bone-related cells is currently unexplored. In terms of translational potential, novel recent insights into biology of RBPs nominate this class of molecules as novel potential targets given their newly discovered multifunctional role in disease pathomechanisms [25].

Central to this study was the dissection of the transcriptional landscape of the compendium of 180 genes encoding RBPs in bone-forming osteoblasts versus bone-resorbing osteoclasts. We applied a multi-modular systems biology-based approach to answer various research questions. Compendium-wide integrative analyses were performed using the GENEVESTIGATOR platform and the Ingenuity Pathway Analysis software. Tracing those 180 genes in osteoblasts and osteoclasts, analyzing their transcriptional regulation, and reconstructing the gene networks allowed us to nominate promising candidates as novel regulators of bone homeostasis. Furthermore, osteoblast-specific and osteoclast-specific signatures were dissected.

## 2. Results

### 2.1. The Transcriptional Landscape of Genes Encoding 180 RBPs in Osteoblasts and Osteoclasts and Follow-Up Comparative Analysis

**Figure 1:**
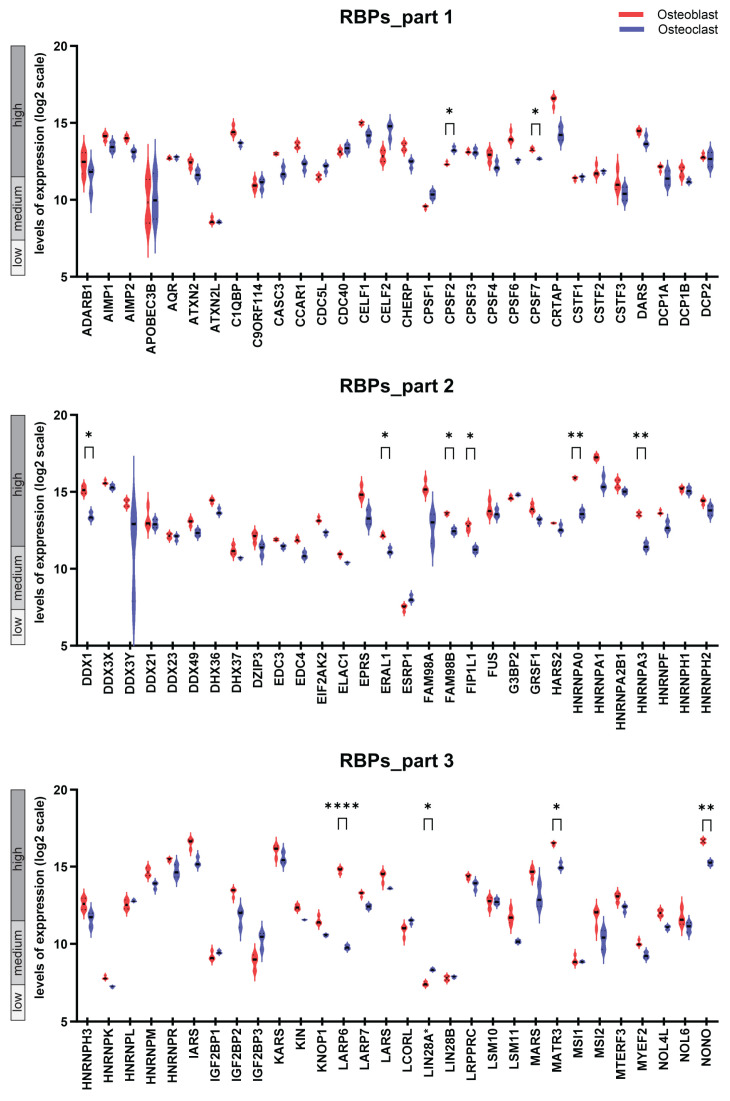

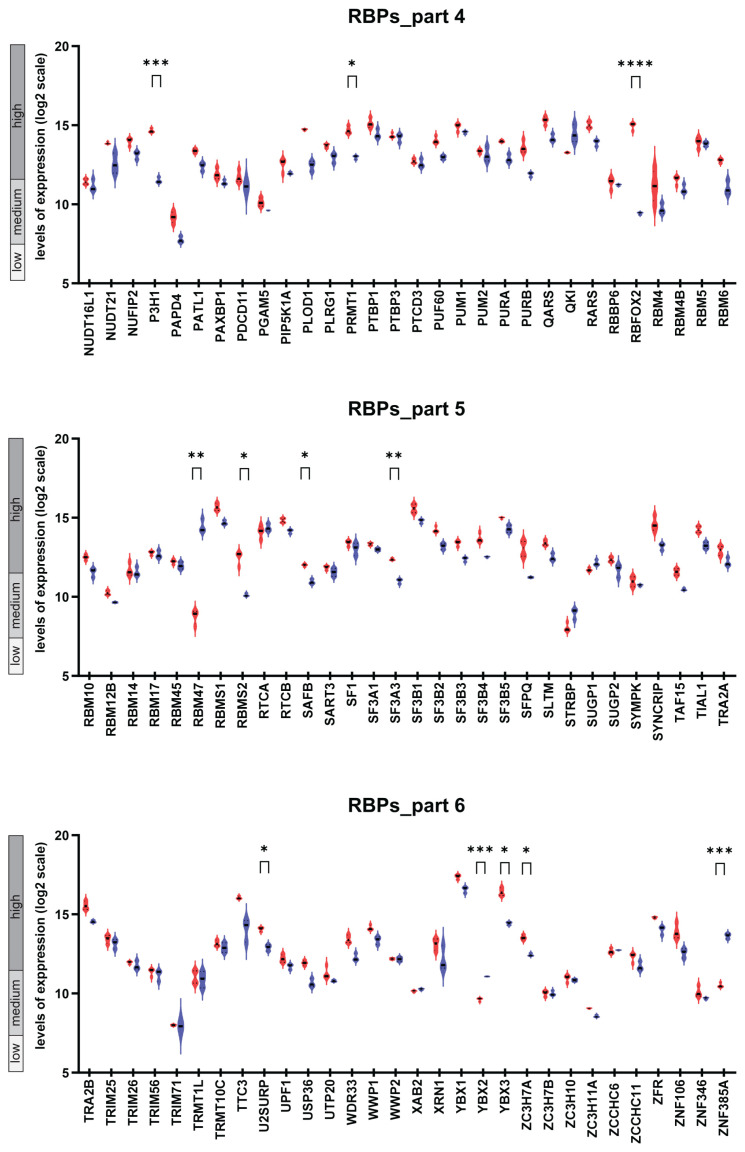
The expression patterns of genes encoding 180 RBPs in osteoblasts and osteoclasts. GENEVESTIGATOR-based analysis was performed to extract the expression levels of the genes encoding the 180 RBPs in osteoblasts and osteoclast. Analysis was performed on the basis of the Affymetrix Human Genome U133 Plus 2.0 Array platform; specific filters were set to define osteoblasts (n = 4, derived from the GSE12264 data set [26]) and osteoclasts (n = 3, derived from the GSE63009 data set [27]). For both cell types, only untreated/mock treated samples were included. Box-plots represent the expression levels of the individual genes encoding RBPs. The levels of expression are given as log2 transformed values and are sub-divided into low, medium, and high expression according to GENEVESTIGATOR. Color code: red, osteoblasts; blue, osteoclasts. Group comparison was performed using *t*-test; the correction for multiple testing was performed using the Bonferroni–Holm method. The *p*-value upon Bonferroni–Holm correction are indicated: * *p* ≤ 0.05, ** *p* ≤ 0.01, *** *p* ≤ 0.001, and **** *p* ≤ 0.0001; only significant *p*-values are indicated. *LIN28A* showed low mRNA expression levels in both cell types; the biological relevance of this level of expression needs further validation. The data were assessed and extracted from GENEVESTIGATOR on 31 August 2021.

**Figure 2:**
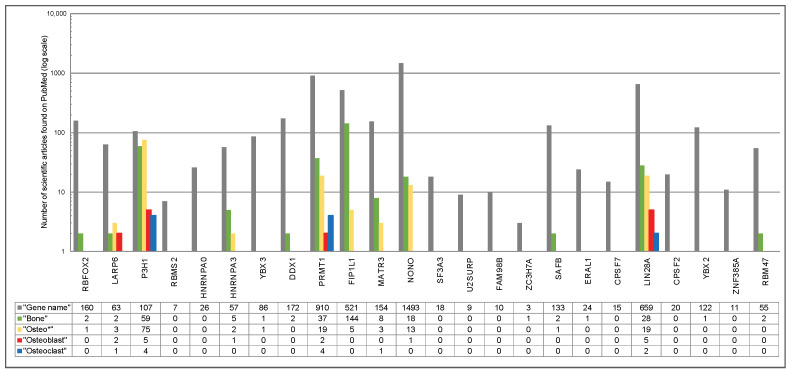
Existing knowledge on the 24 differentially expressed genes. PubMed-based search was conducted first for the gene name alone and then using the combination of keywords “Gene Name” (such as *RBFOX2*) AND the indicated term including “Bone”, “Osteo *”, Osteoblast”, and “Osteoclast” (assessed on 5 November 2023). The outcome is shown by a bar chart; color code: dark gray, “Gene name”; green, “Bone”; yellow, “Osteo *”; red, “Osteoblast”; blue, “Osteoclast”. The number of scientific articles found on PubMed for each search condition is indicated using a log scale. Genes were classified as “limited knowledge in bone homeostasis” if for the search terms “Bone”, “Osteo *”, Osteoblast”, and “Osteoclast” ≤ 2 publications were found.

In the first analytical module, we dissected the transcriptional abundance of genes encoding 180 RBPs in osteoblasts and osteoblasts (Figure 1). As a result, for each cell type, genes can be sub-divided into three categories: (i) those found to be not expressed or expressed on the low level, (ii) genes with moderate expression, and (iii) genes with high levels of expression. Furthermore, comparative analysis of the expression patterns of the compendium of 180 genes encoding RBPs revealed that 124 genes were differentially expressed when comparing the expression levels in osteoblasts and osteoclasts (Table S1). After performing the Bonferroni–Holm correction for multiple testing, the number of differentially expressed genes was 24 (Table 1). Next, we sub-divided the set of 24 differentially expressed genes into two groups: genes that were significantly higher expressed in osteoblasts (n = 19) and those that were significantly higher expressed in osteoclasts (n = 5) (Table 1). Finally, based on the information extracted from the comprehensive literature search (Figure 2) we could sub-divide the genes into two categories: those genes that are known to have an impact into bone homeostasis and genes for which very limited knowledge is currently available. From the genes that showed higher expression in osteoblasts, the first category included *RBFOX2*, *LARP6*, *P3H1*, *HNRNPA3*, *PRMT1*, *FIP1L1*, *MATR3*, and *NONO*, and the second category included *RBMS2*, *HNRNPA0*, *YBX3*, *DDX1*, *SF3A3*, *U2SURP*, *FAM98B*, *ZC3H7A*, *SAFB*, *ERAL1*, and *CPSF7*. For genes that showed higher expression in osteoclasts, the gene with known function in bone metabolism was *LIN28A* and the genes with limited knowledge were *CPSF2*, *YBX2*, *ZNF385A*, and *RBM47*.

### 2.2. Regulation on the Gene Expression Level of 180 RBPs upon Various Treatment Conditions

In the second analytical module, we dissected the changes in the expression levels of 180 genes encoding RBPs upon treatment with various prominent bone biology-associated agents. This included the treatment of (i) osteoblasts with 10^−7^ M of dexamethasone (early time point 2 h, late time point 24 h; data derived from the GSE10311 data set [28]); (ii) osteoblasts with 10^−4^ mg/mL of bone morphogenic protein (BMP)-2 (early time point 2 h, late time point 24 h; data derived from the GSE10311 data set [28]); (iii) mononuclear cells (MNCs) as osteoblast precursors with differentiation and mineralization medium containing 10 mM β-glycerophosphate (early time point 24 h, intermediate time point 7 d, late time point 10–14 d; data derived from the GSE12264 data set [26]); and (iv) osteoclasts with bisphosphonates, more precisely with 100 nM alendronate or 100 nM risedronate, both for 8 d (GSE63009 data set [27]). To define those genes, which were significantly up- or down-regulated under the indicated conditions, we used the following filter conditions: *p*-value ≤ 0.05 and fold change ≥ |1.5|.

**Figure 3:**
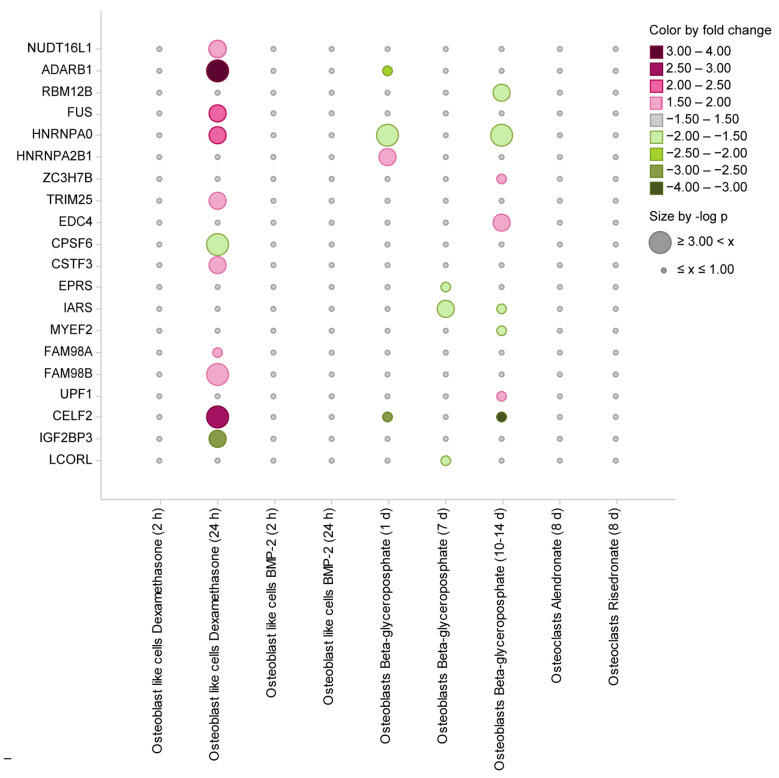
Up-regulation and down-regulation of genes encoding RBPS upon various treatment conditions. Bubble plot shows the 20 genes that were found to be significantly up- or down-regulated (*p*-value ≤ 0.05 and fold change ≥ |1.5|). The cell types, types of treatment and treatment durations are indicated. The success of the treatments was shown within the corresponding original publications; this includes a validation of the microarray results by quantitative real-time PCR [26,27,28]. The color indicates the strength and direction of the regulation (magenta, up-regulation; green, down-regulation). Dot size is proportional to the −log *p*-value. The data were assessed and extracted from GENEVESTIGATOR on 1 September 2021. The data were visualized using Spotfire software on 5 May 2022.

The analysis revealed 20 genes to be significantly up- or down-regulated in response to at least one of the above treatment conditions (Figure 3 and Table S2). The analysis performed in this module allowed to define biological contexts in which a change in the expression level of one or more genes encoding RBPs occurs. Based on the obtained results, the genes were grouped based on their behavior upon treatment. The strongest change in expression levels across the analyzed RBPs was detected after the 24 h dexamethasone treatment, resulting in n = 11 genes with significant change in their expression levels, out of which n = 9 were up-regulated (*NUDT16L1*, *ADARB1*, *FUS*, *HNRNPA0*, *TRIM25*, *CSTF3*, *FAM98A*, *FAM98B*, *CELF2*) and n = 2 were down-regulated (*CPSF6*, *IGF2BP3*). In contrast, short dexamethasone treatment (2 h) showed no significant changes in expression levels of analyzed RBPs. The second group represents the genes (n = 8) with the long-term (10–14 d) ß-glycerophosphate treatment, where n = 3 genes were up-regulated (*ZC3H7B*, *EDC4*, *UPF1*) and n = 5 were down-regulated (*RBM12B*, *HNRNPA0*, *IARS*, *MYEF2*, *CELF2*). The third group represents genes with short-term (1 d) ß-glycerophosphate treatment, which includes n = 4 genes, out of which n = 1 was up-regulated (*HNRNPA2B1*) and n = 3 were down-regulated (*ADARB1*, *HNRNPA0*, *CELF2*). The fourth group consists of n = 3 down-regulated genes upon intermediate (t = 7 d) ß-glycerophosphate treatment (*EPRS*, *IARS*, *LCORL*) and no genes that show up-regulation. Other treatments (BMP-2, bisphosphonates) and treatment durations (dexamethasone early, 2 h) showed no significant changes in expression levels of the compendium of RBPs. The potential long-term effects of BMP-2 on the expression of genes encoding RBPs could not be addressed as this agent is known to be applied in vitro to osteoblasts in short-term [29]. Furthermore, we evaluated the data from the perspective of individual genes and their response to the different treatments and/or treatment durations. In this case, *HNRNPA0* showed significant up-regulation upon late dexamethasone treatment (2.18-fold change) at the same time showing down-regulation upon ß-glycerophosphate both short- and long-term treatment with comparable fold changes of −1.56 and −1.51, respectively. *CELF2* has shown changes in expression levels under the same treatment conditions with even stronger response: up-regulation upon dexamethasone late treatment (2.91-fold) and down-regulation with ß-glycerophosphate short- and long-term (−2.77-fold and −3.17-fold, respectively). The strongest response was detected for *ADARB1*, showing 3.63-fold up-regulation after long (24 h) dexamethasone treatment (whereas upon short treatment with ß-glycerophosphate we detected a −2.42-fold down-regulation of the expression level), followed by *CELF2* with 2.91-fold up-regulation after dexamethasone. Other remaining genes are mostly affected by only one type of treatment: n = 8 genes only by dexamethasone late (*NUDT16L1*, *FUS*, *TRIM25*, *CPSF6*, *CSTF3*, *FAM98A*, *FAM98B*, *IGF2BP3*) and n = 8 genes by only ß-glycerophosphate with different treatment durations (*RBM12B*, *HNRNPA2B1*, *ZC3H7B*, *EDC4*, *EPRS*, *MYEF2*, *UPF1*, *LCORL*), with the only exception being *IARS*, which showed significant, yet comparable down-regulation under both intermediate (−1.71-fold) and late (−1.81-fold) ß-glycerophosphate treatment.

Overall, the data demonstrate that dexamethasone tends to promote up-regulation (nine genes), whereas ß-glycerophosphate rather promotes down-regulation (eight genes) of genes encoding RBPs.

**Figure 4:**
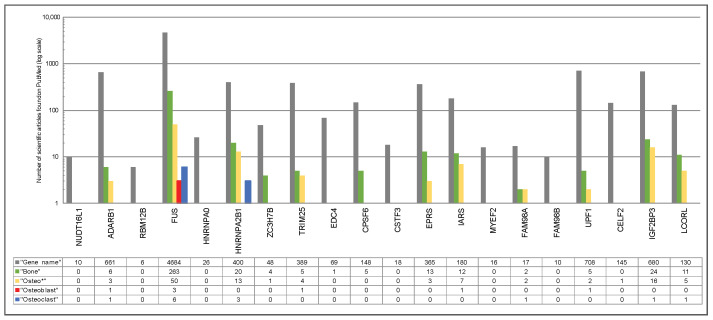
Existing knowledge on the up-regulated and down-regulated genes. PubMed-based search was conducted first for the gene name alone and then using the combination of keywords “Gene Name” (such as *NUDT16L1*) AND the indicated term including “Bone”, “Osteo *”, Osteoblast”, and “Osteoclast” (assessed on 14 November 2023). The outcome is shown by a bar chart; color code: dark gray, “Gene name”; green, “Bone”; yellow, “Osteo *”; red, “Osteoblast”; blue, “Osteoclast”. The number of scientific articles found on PubMed for each search condition is indicated using a log scale. Genes were classified as “limited knowledge in bone homeostasis” if for the search terms “Bone”, “Osteo *”, Osteoblast”, and “Osteoclast” ≤ 2 publications were found.

To determine whether the defined 20 genes encoding RBPs are known bone-related regulators or represent unknown molecules in the field of bone research, a comprehensive literature research was performed. The performed literature research revealed twelve genes (*ADARB1*, *FUS*, *HNRNPA2B1*, *ZC3H7B*, *TRIM25*, *CPSF6*, *EPRS*, *IARS*, *FAM98A*, *UPF1*, *IGF2BP3*, *LCORL*), which were previously reported to play a role in bone metabolism and eight genes (*NUDT16L1*, *RBM12B*, *HNRNPA0*, *EDC4*, *CSTF3*, *MYEF2*, *FAM98B*, *CELF2*) with no/limited knowledge regarding their involvement in bone-related processes (Figure 4).

When comparing the genes dissected within the first two analytical modules—genes with differential expression in osteoblasts and osteoclasts and genes with up- or down-regulation upon different treatments of osteoblasts and osteoclasts—we found two genes in the overlap: *HNRNPA0* and *FAM98B*.

### 2.3. Difference in the Expression Pattern of the 180 Genes Encoding RBPs in Osteoblasts and Osteoclast Dissected by Hierarchical Clustering

The third analytical module focuses on the clustering of the compendium of 180 genes encoding RBPs. For this, the Hierarchical Clustering Tool in GENEVESTIGATOR was used and clustering was performed both across samples attributed to osteoblasts and osteoclasts and across the 180 genes.

**Figure 5:**
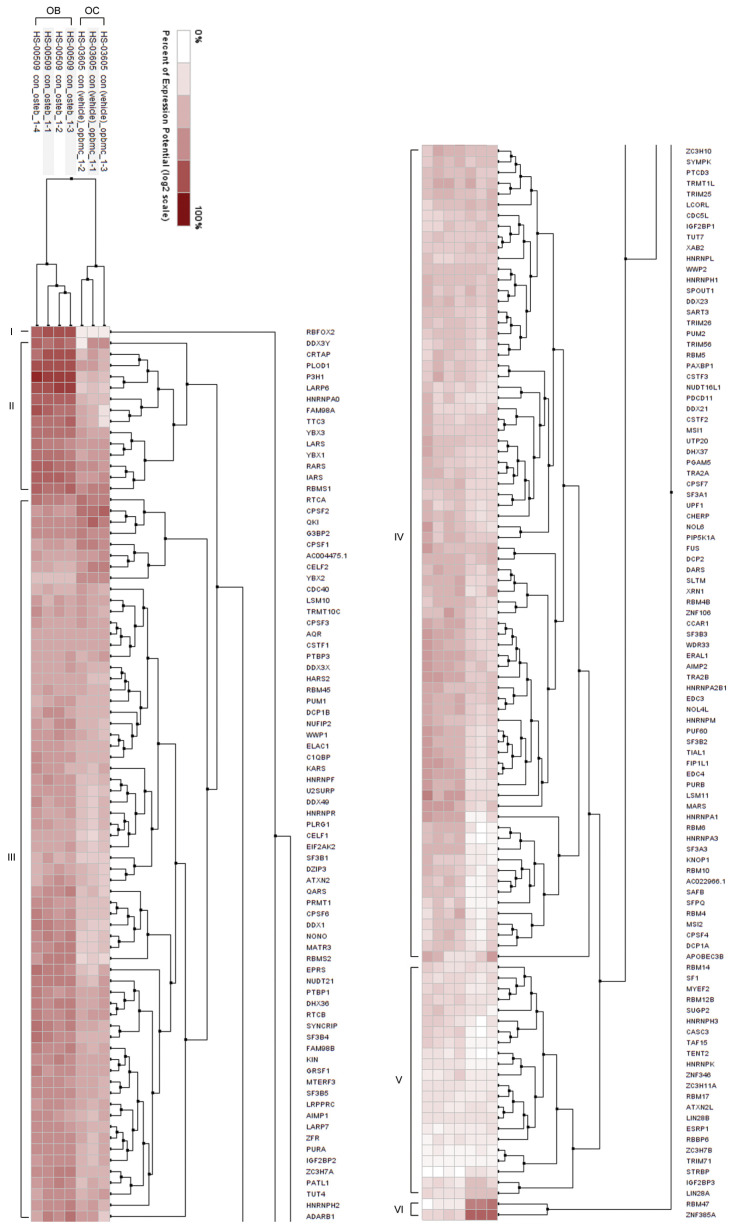
Hierarchical clustering of 180 genes encoding RBPs in osteoblasts and osteoclasts. Two-way hierarchical clustering based on Euclidean distance was performed on the expression of 180 genes across individual samples attributed to osteoblasts (n = 4, derived from the GSE12264 data set [26]) and osteoclasts (n = 3, derived from the GSE63009 data set [27]). Six sub-clusters (I–VI) are indicated. OC, osteoclasts; OB, osteoblasts. The data were assessed and extracted from GENEVESTIGATOR on 11 October 2021.

The samples were sub-divided in two main clusters: one comprising samples attributed to osteoclasts and one comprising samples attributed to osteoblasts (Figure 5). Visual analysis revealed a profound difference in the expression pattern across the 180 RBPs in osteoblasts and osteoclasts, meaning that those two cell types are characterized by differences in the repertoire of RBPs. The clustering across the 180 genes revealed a clear sub-division into six sub-clusters. Out of the six sub-clusters, five sub-clusters (Cluster I–V) showed overall higher mean expression values (calculated across all genes in a cluster) in osteoblasts (Table 2). In contrast to that, the cluster VI was characterized by higher expression levels in osteoclasts; this sub-cluster includes two genes—*RBM47* and *ZNF385A* (Table 2). Overall, the data indicate that the genes encoding the compendium of 180 RBPs are more dominant in bone-forming osteoblasts than in bone-resorbing osteoclasts.

### 2.4. Osteoblast- and Osteoclast-Specific Gene Signatures

The fourth analytical module does not focus exclusively on RBPs but aimed at identifying the cell type-specific gene signatures for our main cell types of interest—the osteoblasts and the osteoclasts. We then verified whether the genes encoding the compendium of RBPs are part of those signatures. As the analytical solution, we used the Gene Search Tool within the GENEVESTIGATOR platform to identify genes specifically expressed in a predefined biological context, which, in the current study, are the two cell types of interest. To dissect such gene signatures within GENEVESTIGATOR, a compendium-wide analysis was performed comparing the transcriptomic finger print of the cell type of interest against a great variety of other cell types and tissue types (n = 777 anatomical parts). As a result, a specific signature was defined, which included the genes highly expressed in the cell type of interest and, at the same time, showed no/low expression for all remaining cell types.

**Figure 6:**
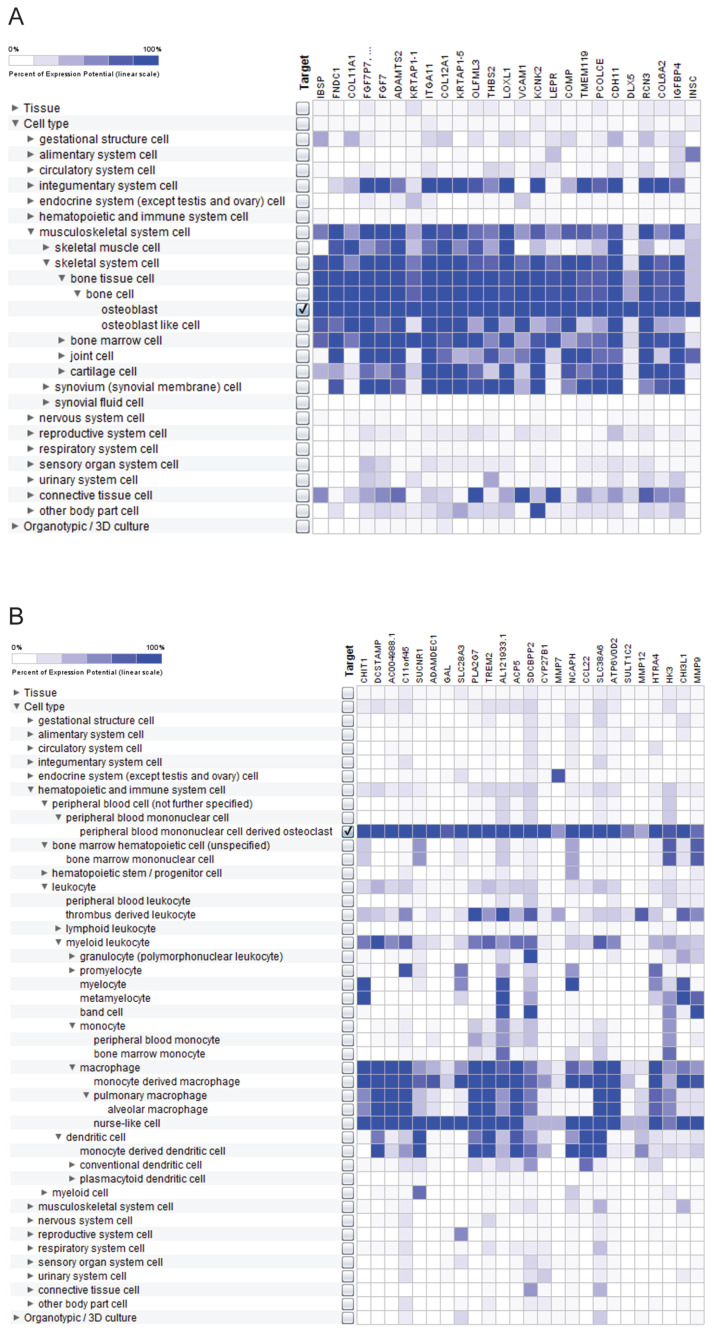
The 25-gene osteoblast-specific gene signature and the 25-gene osteoclast-specific gene signature. Shown are the top 25 genes, which compose the specific gene signature for osteoblasts (**A**) and osteoclasts (**B**). The cell type of interest, for which the specific signature was dissected, is indicated by the checkmark. The expression pattern in other cell types/systems is indicated according to the color code in blue. The analysis was performed across the compendium of 777 anatomical parts on the basis of the Affymetrix Human Genome U133 Plus 2.0 Array platform. In (**A**), the name “FGF7P7, …” is indicative for the *FGF7P-1 to 8* pseudogenes. The data were assessed and extracted from GENEVESTIGATOR on 18 February 2022.

Applying the described strategy, we dissected the 25-gene osteoblast-specific signature (Figure 6A, Table S3) and the 25-gene osteoclast-specific signature (Figure 6B, Table S4). For the osteoblast-specific gene signature, most of the identified genes, besides strong expression in osteoblasts, were found to be expressed at high levels in the entire musculoskeletal system, which osteoblasts are also part of. Additionally, an overlap was found with the integumentary system with twelve out of twenty-five genes showing comparably high expression and four additional genes showing moderate expression. For all other cell types/systems included into the analysis, no/low expression of the signature genes was detected. Within the 25-gene osteoblast-specific signature, we did not find genes that are part of the compendium encoding the 180 RBPs. The 25-gene osteoclast-specific signature showed a strong specificity for the osteoclasts and, in contrast to the osteoblast-specific signature, did not overlap with the overall musculoskeletal system. In turn, ten genes composing the signature showed expression at high levels in macrophages. Regarding genes encoding RBPs, no gene of the compendium was identified within the 25-gene osteoclast-specific signature.

**Figure 7:**
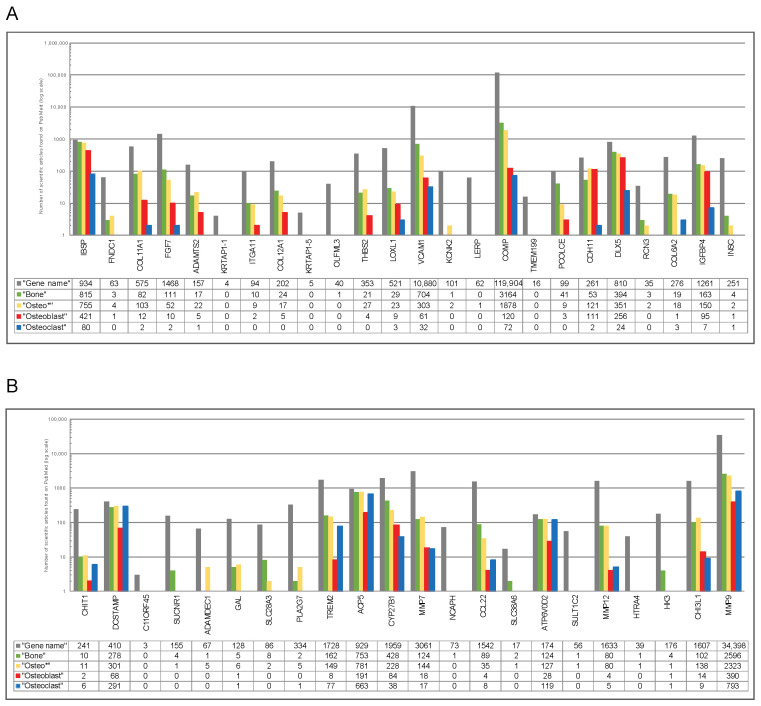
Existing knowledge on the genes comprising the 25-gene osteoblast-specific gene signature and the 25-gene osteoclast-specific gene signature. PubMed-based search was conducted first for the gene name alone and then using the combination of keywords “Gene Name” (such as *IBSP*) AND the indicated term including “Bone”, “Osteo *”, Osteoblast”, and “Osteoclast” (assessed on 9 November 2023) for the 25-gene osteoblast-specific gene signature (**A**) and the 25-gene osteoclast-specific gene signature (**B**). The outcome is shown by a bar chart; color code: dark gray, “Gene name”; green, “Bone”; yellow, “Osteo *”; red, “Osteoblast”; blue, “Osteoclast”. The number of scientific articles found in PubMed for each search condition is indicated using a log scale. Genes were classified as “limited knowledge” in osteoblasts (**A**) or osteoclasts (**B**) if for the search terms “Osteoblast” or “Osteoclast”, respectively, ≤2 publications were found. The transcripts *AC004988.1*, *AL121933* and the pseudogenes *FGF7P-1 to 8* and *SDCBPP2*, where no information is available in NCBI Gene, were excluded from the analysis.

Given the novelty of findings, a systematic literature search combined with information derived from NCBI Gene (Tables S3 and S4) was performed to determine whether the genes composing the specific signatures belong to known players in the corresponding bone cells or whether new promising candidates were uncovered by the applied integrative analysis. For the osteoblast-specific signature, the performed literature mining (Figure 7A) revealed that thirteen genes (*IBSP*, *COL11A1*, *FGF7*, *ADAMTS2*, *COL12A1*, *THBS2*, *LOXL1*, *VCAM1*, *COMP*, *PCOLCE*, *CDH11*, *DLX5*, *IGFBP4*) belong to the known markers of osteoblasts, out of which the most well studied (n > 100 published articles) were four genes, namely *IBPS*, *COMP*, *CDH11*, and *DLX5*. While in contrast, eleven genes (*FNDC1*, *KRTAP1-1*, *ITGA11*, *KRTAP1-5*, *OLFML3*, *KCNK2*, *LERP*, *TMEM199*, *RCN3*, *COL62A*, *INSC*) represent novel promising candidate genes for osteoblasts. For the osteoclast-specific signature, the literature mining (Figure 7B) revealed that eleven genes (*CHIT1*, *DCSTAMP*, *TREM2*, *ACP5*, *CYP27B1*, *MMP7*, *CCL22*, *ATP6V0D2*, *MMP12*, *CHI3L1*, *MMP9*) were known markers, out of which four genes (*DCSTAMP*, *ACP5*, *ATP6V0D2*, *MMP9*) were found to be mentioned in n > 100 articles. At the same time, eleven genes (*C11ORF45*, *SUCNR1*, *ADAMDEC1*, *GAL*, *SLC28A3*, *PLA2G7*, *NCAPH*, *SLC38A6*, *SULT1C2*, *HTRA4*, *HK3*) were identified as novel promising candidate molecules for osteoclasts.

### 2.5. Gene Network Reconstruction and the Nomination of Genes Encoding RBPs as Promising Candidates in Osteoblasts and Osteoclasts

**Figure 8:**
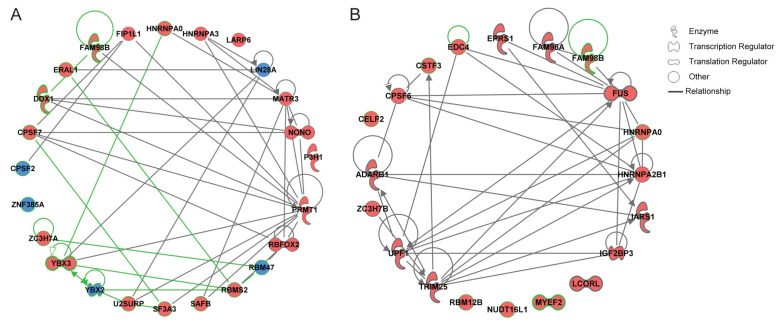
The reconstructed gene networks and the promising candidate RBPs for osteoblasts and osteoclast. IPA software was used to reconstruct the gene networks on the basis of the 24 differentially expressed RBP genes (**A**) and the 20 RBP genes that were found to be significantly up- or down-regulated (**B**). A circular plot view was used for visualization. The types of molecules encoded by the corresponding genes are indicated in the figure legend. Lines display the IPA-identified associations between molecules; color code: red fill, genes encoding RBPs attributed to osteoblasts; blue fill, genes encoding RBPs attributed to osteoclasts; green outline, genes encoding RBPs identified as novel promising candidates. Insert: the IPA-based description of symbols and relationships. The data were assessed and extracted from IPA software on 22 and 23 April 2024.

The fifth analytical module aimed at analyzing the information gained in the first two modules in the context of available knowledge to obtain an overview on how the genes are interconnected. For this, the 24 differentially expressed genes obtained on the basis of the comparison of the transcriptional levels of RBPs in osteoblasts versus osteoclasts (Table 1) and the 20 genes that were found to be significantly up- or down-regulated upon various treatment conditions (Figure 3 and Table S2) were both imported into the Ingenuity Pathway Analysis (IPA) tool for gene network reconstruction (Figure 8). We used the circular plot view for data visualization. Genes encoding RBPs that were found, based on the data obtained in our study, to be linked to osteoblasts were highlighted in red, and those to osteoclasts in blue (Figure 8). Furthermore, we incorporated the findings obtained by the comprehensive literature search to the reconstructed networks; thus, those genes that were proposed by us as promising candidates given no/limited knowledge available regarding their role in bone metabolism were highlighted by a green outline (Figure 8).

For the 24 differentially expressed genes (Figure 8A), we found interconnections among the majority of genes composing the network with only four genes (*ZNF385A*, *CPSF2*, *LARP6*, and *P3H1*) showing no interconnections. Among the fifteen genes that were nominated as promising candidates, thirteen represent an integral part of the gene network and second do not show gene–gene associations.

Among the 20 genes that were found to be significantly up- or down-regulated (Figure 8B), fifteen formed an interconnected gene network and five (*CELF2*, *LCORL*, *MYEF2*, *NUDT16L1*, *RBM12B*) were not linked to each other. Regarding the eight genes that were nominated as promising candidates, four are part of the gene network, while for the reaming four genes the IPA-based analysis did not identify known gene–gene associations.

## 3. Discussion

This study used a comprehensive systems biology-based approach for the analysis of transcriptomic data sets with the focus given to bone-forming osteoblasts and bone-resorbing osteoclasts and the compendium of 180 genes encoding RBPs. The integrative multi-modular dissection of the transcriptional landscape was performed using the state-of-the art analytical solutions GENEVESTIGATOR and the IPA software.

Within the first module, the genes that showed differential expression between osteoblasts and osteoclasts were dissected. These consist of molecules known to be linked to bone metabolism and novel genes that we propose as promising candidates.

With respect to osteoblasts, we specifically highlight *RBFOX2* that was found by us to show the strongest difference in expression between osteoblasts and osteoclasts, with high expression levels found in osteoblasts. *RBFOX2*, with the full name being RNA-binding protein fox-1 homolog 2, is an RBP that plays a critical role in regulating the alternative splicing of pre-mRNAs [30]. The potential involvement of *RBFOX2* in the process of bone remodeling is an area of ongoing research; thus far, studies showed a link between *RBFOX2* and embryonic bone development. The mutation of *Rbfox2* in mouse embryos has led to the development of cleft palate and severe craniofacial abnormalities, whereas a deletion led to neonatal lethality [31]. Furthermore, there is evidence for the impact of RBFOX2 protein on calcium metabolism and the deposition of calcium hydroxyapatite crystals in the rotator cuff, leading to calcific tendinopathy. Although the exact pathogenesis of increased calcium deposition is not fully understood, the study by Cho et al. indicates a link between the RBFOX2 subcellular localization and the disease development [32]. We propose to further study the impact of *RBFOX2* on the cellular level specifically in osteoblasts. The next gene on the list for osteoblasts is *LARP6*. *LARP6* encodes a protein known as La ribonucleoprotein domain family member 6 that is a part of the larger group of RBPs involved in cellular processes primarily related to RNA metabolism and post-transcriptional regulation. One of the well-studied functions of LARP6 protein is its role in stabilizing collagen mRNA, particularly collagen type I [33]. In the context of bone tissue, collagen is synthesized mainly by osteoblasts during bone formation and forms a crucial organic component of the extracellular matrix [34]. The dysregulation of collagen synthesis and its posttranslational modifications may have a role in the pathogenesis of various diseases with compromised bone strength, such as osteoporosis [35]. In contrast to the known link of *LAPR6* to cancer progression [36], the direct association between LARP6 function and bone metabolism has not yet been elucidated in detail. Our study brings attention to *LARP6* as potentially important player in bone metabolism linked to osteoblasts. Hierarchical clustering reveled *RBFOX2* to be not connected to other genes encoding RBPs as it comprises a single gene sub-cluster, whereas *LAPR6* showed a close gene–gene association with *P3H1*, the gene found in our study at the third position within the osteoblasts-attributed genes. To specifically highlight is the novelty of this finding, as within the reconstructed gene network these two genes did not show an interconnection with each other or any other gene from the 24 differentially expressed genes defined in our study. Of note, similar to LAPR6, the P3H1 protein was shown to be involved in the post-translational modification of collagen type 1 and is associated with collagen-related connective tissue disorders, in particular osteogenesis imperfecta type VIII [37]. Thus, our data are suggestive for not-yet-described biological associations between *LAPR6* and *P3H1*.

Within the second category that covers genes with the limited knowledge available thus far, of particular interest is *RMBS2*, which is found on the fourth position in the list of genes attributed to osteoblasts. In contrast to the top three genes—*RBFOX2*, *LARP6*, and *P3H1*—for *RMBS2*, there is thus far no known link to bone metabolism. However, the puzzling information is suggestive of its indirect impact on bone turnover. Thus, a study by Sun et al., in the field of breast cancer, demonstrated that RBMS2 shows anti-proliferative effects and acts as tumor suppressor by stabilizing p21 mRNA and thus increasing p21 protein levels. [38]. In the light of bone turnover, p21 was shown to be associated with bone healing and fracture repair as demonstrated in p21−/− knockout mice [39,40]. What is particularly noteworthy is that the gene network reconstruction performed in our study revealed a strong interconnection of RMBS2 with additional RBPs nominated by us as novel promising candidates. Overall, the data are indicative for the impact of *RMBS2* on bone metabolism; however, its direct role in osteoblasts needs to be elucidated.

Regarding genes encoding RBPs that were found to be linked to osteoclasts, we find on the top position *RBM47*, which showed the strongest differential expression. In *rbm47* knockdown studies in zebrafish, *rbm47* was shown to play a crucial role in embryonic head development [41]. From the list of genes that were found to be linked to osteoclasts, *RBM47* was the only one with a known function in bone development thus far. From the genes encoding RBPs with limited knowledge available, we would like to discuss *ZNF385A*, the second top gene linked to osteoclasts. ZNF385A belongs to the C2H2-type zinc finger protein family, characterized by the presence of multiple zinc finger domains, which are crucial for binding to specific DNA sequences, enabling zinc finger proteins to act as transcription factors [42,43]. However, ZNF385A also shows RNA-binding activity. By this mode of action, ZNF385A was shown to be involved in processes related to cell cycle control and cancer progression [44]. What is particularly noteworthy is the fact that the cluster VI, obtained on the basis of hierarchical clustering, was characterized by higher expression levels of two genes encoding RBPs in osteoclasts, and those are *RBM47* and *ZNF385A*. Thus, the cumulative data from module one and module three strongly interrelate those two RBPs as potentially important players in osteoclasts. This is a novel finding, as the IPA-based gene network reconstruction, utilizing the available knowledge, does not reveal any associations between *ZNF385A* and *RBM47* or any other gene from the 24 differentially expressed genes.

Within the second analytical module, we dissected those genes that were regulated on the mRNA level upon various treatment conditions. One of the major findings is the identification of dexamethasone among the tested agents as the strongest inducer of up-regulation of RBPs mRNA levels. This effect was only observed for the long-term (24 h) treatment with dexamethasone, but not for the short-term (2 h) stimulation. This could be associated with the general mechanism of action of dexamethasone. Dexamethasone belongs to corticosteroids, which act by binding to the intracellular glucocorticoid receptor and initiating the transcription of the target genes. Corticosteroid’s mechanism of action is considered long-term but slow-acting, as the initiated transcription process and consecutive changes in gene expression and/or protein synthesis could require hours for the effect to fully unfold [45]. Additionally, in all cases, where the genes were found to be regulated by both dexamethasone and ß-glycerophosphate, the treatments showed opposing effects on gene expression, indicating their potentially contra-acting roles in bone cell metabolism. Of particular interest is also the fact that the comparison of the genes that were found in the first module with those in the second module revealed only two overlapping genes. This means that the comparative analysis presented here resulted in the discovery of novel osteoblast- and osteoclast-associated candidate genes encoding RBPs, which were not shown to be modulated on the gene expression level by bone-related treatments in previous studies. The strongest effect on the mRNA level was found for *ADARB1*, which was up-regulated by long-term dexamethasone treatment and down-regulated by a short treatment with ß-glycerophosphate in osteoblasts. The *ADARB1* gene encodes the protein Adenosine Deaminase RNA-Specific B1, commonly referred to as ADARB1 or ADAR2. *ADARB1* is responsible for catalyzing the deamination of adenosine to inosine within RNA molecules, effectively changing the RNA sequence of the encoded protein, ultimately impacting protein synthesis and function [46,47]. It is important to highlight that ADARB1 serves as a drug target in various diseases including neurological disorders and cancer [48]. Limited knowledge is available on its role in bone metabolisms; thus, Yu et al. showed that the ablation of *Adar1* decreases bone mass in mice by impairing the function of osteoblasts, including impacts on their differentiation, survival, and proliferation [49]. Our study further accentuates *ADARB1* as an important player in osteoblasts. Our data are thereby suggestive for a more specific/unique role of *ADARB1* in the context of bone turnover, as the analysis performed in the third analytical module did not reveal any close gene–gene associations for *ADARB1*. On the second position, among the most strongly regulated genes encoding RBPs, we found *CELF2*. The gene was up-regulated in osteoblasts upon long-term dexamethasone treatment and down-regulated upon short- and long-term ß-glycerophosphate incubation. The *CELF2* gene encodes the CUGBP Elav-like family member 2, an RBP that interacts with specific RNA sequences and regulates alternative splicing, mRNA stability, and translation [50]. CELF2 functions as a tumor suppressor in multiple cancers including non-small cell lung carcinoma [51] and colon cancer [52]. In contrast to *ADARB1*, *CELF2* is not among the known players in bone metabolism, as revealed by our comprehensive literature search. This is further supported by the findings attributed to the IPA-based gene network reconstruction, where no interaction partners were found for *CELF2*. Overall, we nominate *CELF2* as a promising candidate in bone metabolism.

In addition to the comprehensive analysis of the transcriptional landscape of RBPs in bone cells, our study further focused on the dissection of cell type-specific signatures for the two main cell types of bone homeostasis—osteoblasts and osteoclasts. The 25-gene osteoblasts-specific signature was found to be linked to the entire musculoskeletal system and, unexpectedly, to the integumentary system. The potential agent interrelating the integumentary system to the musculoskeletal system, and more precisely to bone, might be vitamin D3, which is crucial for the skin’s barrier function [53] and is a central player for bone health due to its link to calcium metabolism [54]. The robustness and relevance of the algorithm, that enabled us to identify the 25-gene signature genes, was verified by the fact that among identified genes there are well-known osteoblast-associated molecules such as sialoprotein [55] members of the collagen family [56,57], VCAM1 [58], COMP [59], cadherin 11 [60], and DLX5 [61]. However, in addition to those, the compendium-wide analysis revealed eleven genes that are not currently known as osteoblast-related markers; follow-up investigations, which go beyond the scope of this study, will investigate their relevance in cell-based models and patient specimens. Similar to the osteoblast-specific signature, the integrative analysis revealed the osteoclast-specific signature. Here, the expression pattern was not found to overlap with the entire musculoskeletal system, but showed strong similarity to the one of macrophages. This is in line with previous knowledge as both osteoclasts and macrophages are derived from monocytes as precursors [62,63]. Supporting the relevance of the identified genes, we also found well-known markers, in the case of osteoclasts, such as TREM2 [64], ACP5, also known as TRAP [65], and members of the matrix metallopeptidases [66,67]. Additionally, we dissected eleven genes that were classified by us as novel promising candidate molecules in osteoclasts.

The study has potential limitations given its exploratory nature. Herein, the focus was given to a systems biology-based comprehensive analysis with the nomination of promising candidates as the outcome. Follow-up studies will address the findings in cell-based experiments and by real-time PCR-based analysis, providing further validation.

## 4. Materials and Methods

### 4.1. Comprehensive Analysis of Transcriptomic Data

Comprehensive analysis of transcriptomic data sets was performed using the GENEVESTIGATOR platform (https://genevestigator.com/), which was applied by us previously in various research projects including [68,69]. GENEVESTIGATOR is an analysis platform for publicly available and manually curated transcriptomic data sets allowing to perform a variety of compendium-wide analyses. The focus was given to the set of 180 genes encoding RBPs that were defined in the study by Treiber T et al. [24]. The following studies were included to analyze the cell types of interest and perturbation conditions: GSE12264 [26], GSE63009 [27], and GSE10311 [28]. Key information on GSE data sets is given in Table S5. Detailed descriptions of the individual tools within GENEVESTIGATOR and the filters that were applied are given in the corresponding chapters of Results.

### 4.2. Statistical Analysis and Data Visualization

The log2-transformed expression values for the individual genes comprising the compendium of 180 RBPs were extracted from GENEVESTIGATOR. Group differences were assessed using the *t*-test. SPSS software version 24 was used for statistical analyses; all *p*-values were given as two-sided, and *p* ≤ 0.05 was considered statistically significant. The correction for multiple testing was performed using the Bonferroni–Holm method. Box plots were created using GraphPad Prism software version 10. The bubble plot was created using the Spotfire software (https://www.spotfire.com/) on the basis of the data extracted from GENEVESTIGATOR. Clustering of expression data sets was performed using the Euclidean distance measure within GENEVESTIGATOR based on standard statistical algorithms described in [70].

### 4.3. Gene Network Reconstruction

Gene networks were reconstructed using the Ingenuity Pathway Analysis (IPA) software (https://digitalinsights.qiagen.com/products-overview/discovery-insights-portfolio/analysis-and-visualization/qiagen-ipa/) on the basis of gene lists obtained in the individual analytical modules.

### 4.4. Comprehensive Literature Search

Comprehensive literature search was performed to extract the information on the set of genes that were nominated as promising candidates in the individual analytical modules. PubMed-based search was conducted first for the gene name alone and then using the combination of keywords “Gene Name” AND the indicated term including “Bone”, “Osteo *”, Osteoblast”, and “Osteoclast”. The outcome was shown by bar charts and a summary table was created using Microsoft Excel as part of Microsoft Office Professional Plus 2016.

## 5. Conclusions

This is the first exploratory study that used a comprehensive analysis algorithm to investigate the transcriptional landscape of a compendium of 180 RBPs in bone-forming and bone-resolving cells in the context of bone homeostasis and bone turnover. Furthermore, in this study, the osteoblast- and osteoclast-specific gene signatures were dissected. The obtained findings open new perspectives for future research aiming to investigate the role of particular RNA-binding molecules, identified herein as promising candidates, in cell-based experiments and/or animal models.

## Figures and Tables

**Table 1:** Summary on the expression pattern of the 24 differentially expressed genes encoding RBPs. Listed are RBP encoding genes that showed statistically significant (*p* ≤ 0.05) difference in gene expression between osteoblasts and osteoclasts on the basis of the Bonferroni–Holm corrected *p*-values. Genes are sorted by the difference in the expression value comparing the mean expression values between osteoblasts (OB) and osteoclasts (OC). Color code: red, higher expression in osteoblasts; blue, higher expression in osteoclasts. The genes were then also grouped depending on cell type, in which the selected gene showed higher expression levels (gray mark). * *LIN28A* shows low mRNA expression levels in both cell types; the biological relevance of this level of expression needs further validation.

Gene Name	Expression Level, OB	Mean Value, OB	Difference	Mean Value, OC	Expression Level, OC	*p*-Value
*RBFOX2*	high	14.97	5.54	9.43	medium	<0.0001
*LARP6*	high	14.77	5.00	9.77	medium	<0.0001
*P3H1*	high	14.64	3.12	11.52	medium/high	<0.001
*RBMS2*	high	12.51	2.40	10.11	medium	0.031
*HNRNPA0*	high	15.9	2.33	13.57	high	0.001
*HNRNPA3*	high	13.55	2.09	11.46	medium/high	0.004
*YBX3*	high	16.41	1.99	14.42	high	0.011
*DDX1*	high	15.13	1.72	13.41	high	0.042
*PRMT1*	high	14.69	1.72	12.97	high	0.015
*FIP1L1*	high	12.74	1.50	11.24	medium/high	0.049
*MATR3*	high	16.49	1.46	15.03	high	0.011
*NONO*	high	16.67	1.43	15.24	high	0.007
*SF3A3*	high	12.36	1.37	10.99	medium	0.003
*U2SURP*	high	14.07	1.17	12.9	high	0.037
*FAM98B*	high	13.57	1.12	12.45	high	0.014
*ZC3H7A*	high	13.52	1.08	12.44	high	0.023
*SAFB*	high	12.01	1.07	10.94	medium	0.014
*ERAL1*	high	12.18	1.03	11.15	medium	0.047
*CPSF7*	high	13.28	0.61	12.67	high	0.039
*LIN28A* *	low	7.41	−0.93	8.34	low	0.013
*CPSF2*	high	12.34	−0.94	13.28	high	0.032
*YBX2*	medium	9.62	−1.45	11.07	medium	< 0.001
*ZNF385A*	medium	10.5	−3.13	13.63	high	< 0.001
*RBM47*	medium	8.77	−5.68	14.45	high	0.002

**Table 2:** Comparison of the sub-clusters obtained on the basis of hierarchical clustering. For each gene, the mean value of the expression level for osteoblast-attributed and osteoclast-attributed samples was calculated; next, the overall gene expression levels over all genes in a sub-cluster were assessed for each of the six sub-clusters. The level of expression was classified as low, medium, or high according to GENEVESTIGATOR guidelines. For sub-clusters I–VI the corresponding calculated mean expression values across all genes in a given sub-cluster calculated for osteoblasts (OB) and osteoclasts (OC) are indicated. The symbol “>” indicated higher overall expression in osteoblasts; the symbol “<” indicated higher overall expression in osteoclasts.

Clusters	Expression Level, OB	Mean Value, OB	Comparison	Mean Value, OC	Expression Level, OC
Cluster I	High	14.97	>	9.43	Medium
Cluster II	High	15.54	>	13.43	High
Cluster III	High	13.66	>	13.04	High
Cluster IV	High	12.53	>	11.86	High
Cluster V	Medium	10.06	>	9.82	Medium
Cluster VI	Medium	9.63	<	14.04	High

## Data Availability

The original contributions presented in the study are included in the article/Supplementary Materials; further inquiries can be directed to the corresponding authors.

## Supplementary Materials

The supporting information can be downloaded at https://www.mdpi.com/article/10.3390/ijms251910417/s1.
