# Supplementary material for: RNA-Binding Proteins as Novel Effectors in Osteoblasts and Osteoclasts: A Systems Biology Approach to Dissect the Transcriptional Landscape
Source: Int J Mol Sci. 2024 Sep 27;25(19):10417. doi: 10.3390/ijms251910417 (PMC11476634; doi:10.3390/ijms251910417)
Supplement: Supplementary file 1 [file ijms-25-10417-s001.zip › ijms-3183093-supplementary.pdf]

Table S1. Comparative analysis of gene expression patterns of 180 genes encoding in osteoclasts and osteoblasts. GENEVESTIGATOR-based analysis was performed to extract the expression levels of the genes encoding the 180 RBPs in osteoblasts and osteoclast. Analysis was performed on the basis of the Affymetrix Human Genome U133 Plus 2.0 Array platform; specific filters were set to define osteoblasts (n = 4, derived from the GSE12264 data set) and osteoclasts (n = 3, derived from the GSE63009 data set). The data were assessed and extracted from GENEVESTIGATOR on August 31, 2021. Group comparison was done using T-test; the correction for multiple testing was done using the Bonferroni-Holm method. The p-value and p-value upon Bonferroni-Holm correction are both indicated. Genes are listed alphabetically.

Gene Name	p-value	Bonferroni-Holm corrected p-value
ADARB1	0.15116900	5.74442200
AIMP1	0.01697600	1.37505600
AIMP2	0.00118500	0.15997500
APOBEC3B	0.82985400	4.14927000
AQR	0.76174000	5.33218000
ATXN2	0.01550600	1.31801000
ATXN2L	0.60342200	6.63764200
C1QBP	0.00537400	0.55352200
C9ORF114	0.74116000	5.92928000
CASC3	0.02158400	1.66196800
CCAR1	0.00079700	0.11237700
CDC40	0.21221100	7.42738500
CDC5L	0.00713500	0.67069000
CELF1	0.00308400	0.34540800
CELF2	0.00357800	0.39358000
CHERP	0.00264600	0.29899800
CPSF1	0.00148300	0.19427300
CPSF2	0.00019800	0.03207600
CPSF3	0.92705800	1.85411600
CPSF4	0.04255200	2.51056800
CPSF6	0.00126500	0.16951000
CPSF7	0.00024400	0.03904000
CRTAP	0.00052800	0.07814400
CSTF1	0.44376000	9.31896000
CSTF2	0.99721000	0.99721000
CSTF3	0.14114300	5.78686300
DARS	0.01681400	1.37874800
DCP1A	0.03949800	2.44887600
DCP1B	0.03009400	2.07648600
DCP2	0.46917500	7.97597500
DDX1	0.00026200	0.04165800
DDX21	0.47685700	7.15285500
DDX23	0.44675100	8.93502000
DDX3X	0.02751700	2.03625800
DDX3Y	0.21509600	7.31326400
DDX49	0.00600800	0.60080000
DHX36	0.00214000	0.25466000
DHX37	0.02994600	2.09622000
DZIP3	0.02792400	2.01052800
EDC3	0.00103100	0.14021600
EDC4	0.00039700	0.06074100
EIF2AK2	0.00035200	0.05456000
ELAC1	0.00069000	0.09867000
EPRS	0.00474800	0.50803600
ERAL1	0.00029900	0.04724200
ESRP1	0.00971000	0.87390000
FAM98A	0.00486800	0.51600800
FAM98B	0.00008200	0.01369400
FIP1L1	0.00031300	0.04914100
FUS	0.36384800	9.82389600
G3BP2	0.04064500	2.43870000
GRSF1	0.00525100	0.54610400
HARS2	0.11172300	5.02753500
HNRNPA0	0.00000800	0.00140000
HNRNPA1	0.00060000	0.08760000
HNRNPA2B1	0.02825600	2.00617600
HNRNPA3	0.00002100	0.00361200
HNRNPF	0.00257300	0.29332200
HNRNPH1	0.43569200	9.58522400
HNRNPH2	0.02004400	1.58347600
HNRNPH3	0.02751700	2.06377500
HNRNPK	0.00034400	0.05366400
HNRNPL	0.20171500	7.26174000

HNRNPM	0.00633400	0.61439800
HNRNPR	0.01472400	1.26626400
IARS	0.00247400	0.28945800
IGF2BP1	0.14965400	5.83650600
IGF2BP2	0.00343400	0.38117400
IGF2BP3	0.01565000	1.31460000
KARS	0.12382000	5.32426000
KIN	0.00039000	0.06006000
KNOP1	0.00183400	0.22191400
LARP6	0.00000016	0.00002865
LARP7	0.00053400	0.07849800
LARS	0.00784800	0.72201600
LCORL	0.03931400	2.47678200
LIN28A	0.00007900	0.01327200
LIN28B	0.37048500	9.63261000
LRPPRC	0.01612500	1.33837500
LSM10	0.90223300	2.70669900
LSM11	0.00161000	0.20447000
MARS	0.01027300	0.91429700
MATR3	0.00006600	0.01115400
MSI1	0.58334400	7.58347200
MSI2	0.01903000	1.52240000
MTERF3	0.01198400	1.04260800
MYEF2	0.00230200	0.27163600
NOL4L	0.00096000	0.13152000
NOL6	0.13164000	5.52888000
NONO	0.00003900	0.00666900
NUDT16L1	0.25622800	7.68684000
NUDT21	0.06928000	3.53328000
NUFIP2	0.00551700	0.56273400
P3H1	0.00000100	0.00017800
PAPD4	0.00159700	0.20601300
PATL1	0.00141000	0.18612000
PAXBP1	0.05353400	2.99790400
PDCD11	0.04858600	2.76940200
PGAM5	0.02273100	1.72755600
PIP5K1A	0.06312700	3.34573100
PLOD1	0.00624800	0.61230400
PLRG1	0.00644200	0.61843200
PRMT1	0.00008800	0.01452000
PTBP1	0.03157100	2.11525700
PTBP3	0.47593100	7.61489600
PTCD3	0.34849700	9.75791600
PUF60	0.00076300	0.10834600
PUM1	0.05751400	3.16327000
PUM2	0.40163000	9.23749000
PURA	0.00044900	0.06735000
PURB	0.00065400	0.09483000
QARS	0.00173600	0.21700000
QKI	0.06395500	3.32566000
RARS	0.00095300	0.13151400
RBBP6	0.47892700	6.70497800
RBFOX2	0.00000018	0.00003149
RBM10	0.00249700	0.28965200
RBM12B	0.00250400	0.28796000
RBM14	0.70210100	6.31890900
RBM17	0.44783400	8.50884600
RBM4	0.05872300	3.17104200
RBM45	0.11842000	5.21048000
RBM47	0.00001200	0.00208800
RBM4B	0.03107400	2.11303200
RBM5	0.60328200	7.23938400
RBM6	0.00066100	0.09518400
RBMS1	0.00148500	0.19305000
RBMS2	0.00018800	0.03064400
RTCA	0.37851800	9.08443200
RTCB	0.00170600	0.21495600
SAFB	0.00008400	0.01394400
SART3	0.10540100	4.84844600
SF1	0.14705600	5.88224000
SF3A1	0.00622600	0.61637400
SF3A3	0.00001600	0.00276800

SF3B1	0.00678100	0.64419500
SF3B2	0.00080300	0.11242000
SF3B3	0.00040300	0.06125600
SF3B4	0.00093300	0.12968700
SF3B5	0.00175700	0.21786800
SFPQ	0.00554600	0.56014600
SLTM	0.00203800	0.24456000
STRBP	0.00884100	0.80453100
SUGP1	0.01109800	0.97662400
SUGP2	0.02783300	2.03180900
SYMPK	0.24553800	7.61167800
SYNCRIP	0.00394600	0.43011400
TAF15	0.00041700	0.06296700
TIAL1	0.00160900	0.20595200
TRA2A	0.02151500	1.67817000
TRA2B	0.00175700	0.21611100
TRIM25	0.23477100	7.51267200
TRIM26	0.22073100	7.28412300
TRIM56	0.37780500	9.44512500
TRIM71	0.07782100	3.81322900
TRMT10C	0.19728000	7.29936000
TRMT1L	0.69047700	6.90477000
TTC3	0.04007500	2.44457500
U2SURP	0.00023000	0.03703000
UPF1	0.03609500	2.31008000
USP36	0.00048400	0.07211600
UTP20	0.08101200	3.88857600
WDR33	0.00178900	0.21825800
WWP1	0.00423000	0.45684000
WWP2	0.79724700	4.78348200
XAB2	0.04395100	2.54915800
XRN1	0.07767000	3.88350000
YBX1	0.00138800	0.18460400
YBX2	0.00000200	0.00035200
YBX3	0.00006200	0.01054000
ZC3H10	0.34612400	10.03759600
ZC3H11A	0.00488600	0.51303000
ZC3H7A	0.00013900	0.02279600
ZC3H7B	0.88908500	3.55634000
ZCCHC11	0.03575800	2.32427000
ZCCHC6	0.44987300	8.09771400
ZFR	0.03472600	2.29191600
ZNF106	0.00768300	0.71451900
ZNF346	0.10220800	4.80377600
ZNF385A	0.00000100	0.00017700

Table S2. The 20 genes encoding RBPs that are up-regulated and/or down-regulated upon various treatment conditions. Study GSE number, treatment type, treatment duration and cell type are indicated. The fold changes of up-regulation or down-regulation as well as the corresponding p-values are given. Only statistically significant outcomes (p ≤ 0.05) are included. Color code: red, up-regulated; green, down-regulated. Dexa, Dexamethasone; BMP-2, bone morphogenic protein-2; ß-GP, ß-glycerophosphate.

	Study number	GSE10311				GSE12264			GSE63009	
	Treatment	Dexa	Dexa	BMP-2	BMP-2	ß-GP	ß-GP	ß-GP	Alendronate	Risedronate
	Duration	Early	Late	Early	Late	Short	Intermediate	Late		
Gene Name	Cell type	Osteoblasts	Osteoblasts	Osteoblasts	Osteoblasts	Osteoblasts	Osteoblasts	Osteoblasts	Osteoclasts	Osteoclasts
NUDT16L1	fold change		1.75							
	p-value		0.001							
ADARB1	fold change		3.63			-2.42				
	p-value		< 0,001			0.026				
RBM12B	fold change							-1.66		
	p-value							0.007		
FUS	fold change		2.08							
	p-value		0.002							
HNRNPA0	fold change		2.18			-1.56		-1.51		
	p-value		0.001			0.001		0.001		
HNRNPA2B1	fold change					1.64				
	p-value					0.004				
ZC3H7B	fold change							1.54		
	p-value							0.012		
TRIM25	fold change		1.52							
	p-value		0.003							
EDC4	fold change							1.51		
	p-value							0.004		
CPSF6	fold change		-1.53							
	p-value		< 0,001							
CSTF3	fold change		1.55							
	p-value		0.010							
EPRS	fold change						-1.62			
	p-value						0.018			
IARS	fold change						-1.71	-1.81		
	p-value						0.003	0.022		
MYEF2	fold change							-1.62		
	p-value							0.014		
FAM98A	fold change		1.67							
	p-value		0.011							
FAM98B	fold change		1.69							
	p-value		0.001							
UPF1	fold change							1.59		
	p-value							0.022		
CELF2	fold change		2.91			-2.77		-3.17		
	p-value		< 0,001			0.017		0.013		
IGF2BP3	fold change		-2.91							
	p-value		0.002							
LCORL	fold change						-1.53			
	p-value						0.049			

Table S3. Genes composing the 25-gene osteoblast-specific gene signature. Gene symbol, gene ID, synonyms, full name(s), information on the gene type, and a short description from NCBI Gene are provided.

Symbol	Gene ID	Synonym	Name	Gene type	Description/Summary
IBSP	3381	BSP; BNSP; SP-II; BSP-II	integrin binding sialoprotein	protein coding	The protein encoded by this gene is a major structural protein of the bone matrix. It constitutes approximately 12% of the noncollagenous proteins in human bone and is synthesized by skeletal-associated cell types, including hypertrophic chondrocytes, osteoblasts, osteocytes, and osteoclasts. The only extraskeletal site of its synthesis is the trophoblast. This protein binds to calcium and hydroxyapatite via its acidic amino acid clusters, and mediates cell attachment through an RGD sequence that recognizes the vitronectin receptor. [provided by RefSeq, Jul 2008]
FNDC1	84624	AGS8; FNDC2; MEL4B3; bA243O10.1; dJ322A24.1	fibronectin type III domain containing 1	protein coding	Predicted to act upstream of or within several processes, including cellular response to hypoxia; positive regulation of cardiac muscle cell apoptotic process; and positive regulation of protein phosphorylation. Located in nuclear speck. [provided by Alliance of Genome Resources, Apr 2022]
COL11A1	1301	STL2; COLL6; CO11A1; DFNA37	collagen type XI alpha 1 chain	protein coding	This gene encodes one of the two alpha chains of type XI collagen, a minor fibrillar collagen. Type XI collagen is a heterotrimer but the third alpha chain is a post-translationally modified alpha 1 type II chain. Mutations in this gene are associated with

					type II Stickler syndrome and with Marshall syndrome. A single-nucleotide polymorphism in this gene is also associated with susceptibility to lumbar disc herniation. Multiple transcript variants have been identified for this gene. [provided by RefSeq, Nov 2009]
FGF7P1	387559	HsT283	fibroblast growth factor 7 pseudogene 1	pseudo	-
FGF7P2	394217	FGF7L; PRED3	fibroblast growth factor 7 pseudogene 2	pseudo	-
FGF7P3	654466	KGFLP2	fibroblast growth factor 7 pseudogene 3	pseudo	-
FGF7P4	728195	-	fibroblast growth factor 7 pseudogene 4	pseudo	-
FGF7P5	653453	-	fibroblast growth factor 7 pseudogene 5	pseudo	-
FGF7P6	387628	KGFLP1	fibroblast growth factor 7 pseudogene 6	pseudo	-
FGF7P7	728433	-	fibroblast growth factor 7 pseudogene 7	pseudo	-
FGF7P8	100113421	-	fibroblast growth factor 7 pseudogene 8	pseudo	-
FGF7	2252	KGF; HBGF-7	fibroblast growth factor 7	protein coding	The protein encoded by this gene is a member of the fibroblast growth

					factor (FGF) family. FGF family members possess broad mitogenic and cell survival activities, and are involved in a variety of biological processes, including embryonic development, cell growth, morphogenesis, tissue repair, tumor growth and invasion. This protein is a potent epithelial cell-specific growth factor, whose mitogenic activity is predominantly exhibited in keratinocytes but not in fibroblasts and endothelial cells. Studies of mouse and rat homologs of this gene implicated roles in morphogenesis of epithelium, reepithelialization of wounds, hair development and early lung organogenesis. [provided by RefSeq, Jul 2008]
ADAMTS2	9509	NPI; PNPI; PCINP; PCPNI; PCI-NP; PC I-NP; ADAM-TS2; ADAMTS-2; ADAMTS-3; EDSDERMS	ADAM metallopeptidase with thrombospondin type 1 motif 2	protein coding	This gene encodes a member of the ADAMTS (a disintegrin and metalloproteinase with thrombospondin motifs) protein family. Members of the family share several distinct protein modules, including a propeptide region, a metalloproteinase domain, a disintegrin-like domain, and a thrombospondin type 1 (TS) motif. Individual members of this family differ in the number of C-terminal TS motifs, and some have unique C-terminal domains. The encoded preproprotein is proteolytically processed to generate the mature procollagen N-proteinase. This proteinase excises the N-propeptide of the fibrillar

					procollagens types I-III and type V. Mutations in this gene cause Ehlers-Danlos syndrome type VIIC, a recessively inherited connective-tissue disorder. Alternative splicing results in multiple transcript variants, at least one of which encodes an isoform that is proteolytically processed. [provided by RefSeq, Feb 2016]
KRTAP1-1	81851	HB2A; KAP1.1; KAP1.6; KAP1.7; KAP1.1A; KAP1.1B; KRTAP1A; hKAP1.7; KRTAP1.1	keratin associated protein 1-1	protein coding	This protein is a member of the keratin-associated protein (KAP) family. The KAP proteins form a matrix of keratin intermediate filaments which contribute to the structure of hair fibers. KAP family members appear to have unique, family-specific amino- and carboxyl-terminal regions and are subdivided into three multi-gene families according to amino acid composition: the high sulfur, the ultrahigh sulfur, and the high tyrosine/glycine KAPs. This protein is a member of the high sulfur KAP family and the gene is localized to a cluster of KAPs at 17q12-q21. [provided by RefSeq, Jul 2008]
ITGA11	22801	HsT18964	integrin subunit alpha 11	protein coding	This gene encodes an alpha integrin. Integrins are heterodimeric integral membrane proteins composed of an alpha chain and a beta chain. This protein contains an I domain, is expressed in muscle tissue, dimerizes with beta 1 integrin in vitro, and appears to bind collagen in this form. Therefore, the protein may be involved in

					attaching muscle tissue to the extracellular matrix. Alternative transcriptional splice variants have been found for this gene, but their biological validity is not determined. [provided by RefSeq, Jul 2008]
COL12A1	1303	UCMD2; BTHLM2; EDSMYP; COL12A1L; BA209D8.1; DJ234P15.1	collagen type XII alpha 1 chain	protein coding	This gene encodes the alpha chain of type XII collagen, a member of the FACIT (fibril-associated collagens with interrupted triple helices) collagen family. Type XII collagen is a homotrimer found in association with type I collagen, an association that is thought to modify the interactions between collagen I fibrils and the surrounding matrix. Alternatively spliced transcript variants encoding different isoforms have been identified. [provided by RefSeq, Jul 2008]
KRTAP1-5	83895	KAP1.5; KRTAP1.5	keratin associated protein 1-5	protein coding	This protein is a member of the keratin-associated protein (KAP) family. The KAP proteins form a matrix of keratin intermediate filaments which contribute to the structure of hair fibers. KAP family members appear to have unique, family-specific amino- and carboxyl-terminal regions and are subdivided into three multi-gene families according to amino acid composition: the high sulfur, the ultrahigh sulfur, and the high tyrosine/glycine KAPs. This protein is a member of the high sulfur KAP family and the gene is localized to a cluster of KAPs at 17q12-q21.

					[provided by RefSeq, Jul 2008]
OLFML3	56944	OLF44; HNOEL-iso	olfactomedin like 3	protein coding	This gene encodes a member of the olfactomedin-like gene family which also includes genes encoding noelin, tiarin, myocilin, amassin, optimedin, photomedin, and latrophilin. The encoded protein is a secreted extracellular matrix glycoprotein with a C-terminal olfactomedin domain that facilitates protein-protein interactions, cell adhesion, and intercellular interactions. It serves as both a scaffold protein that recruits bone morphogenetic protein 1 to its substrate chordin, and as a vascular tissue remodeler with pro-angiogenic properties. Alternative splicing results in multiple transcript variants. [provided by RefSeq, Feb 2017]
THBS2	7058	TSP2	thrombospondin 2	protein coding	The protein encoded by this gene belongs to the thrombospondin family. It is a disulfide-linked homotrimeric glycoprotein that mediates cell-to-cell and cell-to-matrix interactions. This protein has been shown to function as a potent inhibitor of tumor growth and angiogenesis. Studies of the mouse counterpart suggest that this protein may modulate the cell surface properties of mesenchymal cells and be involved in cell adhesion and migration. [provided by RefSeq, Jul 2008]

LOXL1	4016	LOL; LOXL	lysyl oxidase like 1	protein coding	This gene encodes a member of the lysyl oxidase family of proteins. The prototypic member of the family is essential to the biogenesis of connective tissue, encoding an extracellular copper-dependent amine oxidase that catalyzes the first step in the formation of crosslinks in collagen and elastin. The encoded preproprotein is proteolytically processed to generate the mature enzyme. A highly conserved amino acid sequence at the C-terminus end appears to be sufficient for amine oxidase activity, suggesting that each family member may retain this function. The N-terminus is poorly conserved and may impart additional roles in developmental regulation, senescence, tumor suppression, cell growth control, and chemotaxis to each member of the family. Mutations in this gene are associated with exfoliation syndrome. [provided by RefSeq, Jan 2016]
VCAM1	7412	CD106; INCAM-100	vascular cell adhesion molecule 1	protein coding	This gene is a member of the Ig superfamily and encodes a cell surface sialoglycoprotein expressed by cytokine-activated endothelium. This type I membrane protein mediates leukocyte-endothelial cell adhesion and signal transduction, and may play a role in the development of artherosclerosis and rheumatoid arthritis. Three alternatively

					spliced transcripts encoding different isoforms have been described for this gene. [provided by RefSeq, Dec 2010]
KCNK2	3776	TREK; TPKC1; TREK1; K2p2.1; TREK-1; hTREK-1c; hTREK-1e	potassium two pore domain channel subfamily K member 2	protein coding	This gene encodes one of the members of the two-pore-domain background potassium channel protein family. This type of potassium channel is formed by two homodimers that create a channel that leaks potassium out of the cell to control resting membrane potential. The channel can be opened, however, by certain anesthetics, membrane stretching, intracellular acidosis, and heat. Three transcript variants encoding different isoforms have been found for this gene. [provided by RefSeq, Jul 2008]
LEPR	3953	OBR; OB-R; CD295; LEP-R; LEPRD	leptin receptor	protein coding	The protein encoded by this gene belongs to the gp130 family of cytokine receptors that are known to stimulate gene transcription via activation of cytosolic STAT proteins. This protein is a receptor for leptin (an adipocyte-specific hormone that regulates body weight), and is involved in the regulation of fat metabolism, as well as in a novel hematopoietic pathway that is required for normal lymphopoiesis. Mutations in this gene have been associated with obesity and pituitary dysfunction. Alternatively spliced transcript variants encoding different isoforms have been described for this gene. It

					is noteworthy that this gene and LEPROT gene (GeneID:54741) share the same promoter and the first 2 exons, however, encode distinct proteins (PMID:9207021).[provided by RefSeq, Nov 2010]
COMP	1311	MED; CTS2; EDM1; EPD1; TSP5; PSACH; THBS5	cartilage oligomeric matrix protein	protein coding	The protein encoded by this gene is a noncollagenous extracellular matrix (ECM) protein. It consists of five identical glycoprotein subunits, each with EGF-like and calcium-binding (thrombospondin-like) domains. Oligomerization results from formation of a five-stranded coiled coil and disulfides. Binding to other ECM proteins such as collagen appears to depend on divalent cations. Contraction or expansion of a 5 aa aspartate repeat and other mutations can cause pseudochondroplasia (PSACH) and multiple epiphyseal dysplasia (MED). [provided by RefSeq, Jul 2016]
TMEM119	338773	OBIF	transmembrane protein 119	protein coding	Involved in positive regulation of bone mineralization; positive regulation of osteoblast differentiation; and positive regulation of osteoblast proliferation. Located in plasma membrane. [provided by Alliance of Genome Resources, Apr 2022]
PCOLCE	5118	PCPE; PCPE1; PCPE-1	procollagen C-endopeptidase enhancer	protein coding	Fibrillar collagen types I-III are synthesized as precursor molecules known as procollagens. These precursors contain amino- and carboxyl-terminal peptide extensions known as N- and C-propeptides,

					respectively, which are cleaved, upon secretion of procollagen from the cell, to yield the mature triple helical, highly structured fibrils. This gene encodes a glycoprotein which binds and drives the enzymatic cleavage of type I procollagen and heightens C-proteinase activity. [provided by RefSeq, Jul 2008]
CDH11	1009	OB; ESWS; CAD11; CDHOB; OSF-4; TBHS2	cadherin 11	protein coding	This gene encodes a type II classical cadherin from the cadherin superfamily, integral membrane proteins that mediate calcium-dependent cell-cell adhesion. Mature cadherin proteins are composed of a large N-terminal extracellular domain, a single membrane-spanning domain, and a small, highly conserved C-terminal cytoplasmic domain. Type II (atypical) cadherins are defined based on their lack of a HAV cell adhesion recognition sequence specific to type I cadherins. Expression of this particular cadherin in osteoblastic cell lines, and its upregulation during differentiation, suggests a specific function in bone development and maintenance. [provided by RefSeq, Jul 2008]
DLX5	1749	SHFM1; SHFM1D	distal-less homeobox 5	protein coding	This gene encodes a member of a homeobox transcription factor gene family similiar to the Drosophila distal-less gene. The encoded protein may play a role in bone development and fracture healing. Mutation in this gene, which is located in a tail-to-tail

					configuration with another member of the family on the long arm of chromosome 7, may be associated with split-hand/split-foot malformation. [provided by RefSeq, Jul 2008]
RCN3	57333	RLP49	reticulocalbin 3	protein coding	Enables calcium ion binding activity. Involved in several processes, including collagen biosynthetic process; positive regulation of peptidase activity; and regulation of protein kinase B signaling. Located in endoplasmic reticulum. [provided by Alliance of Genome Resources, Apr 2022]
COL6A2	1292	UCMD1; BTHLM1; PP3610	collagen type VI alpha 2 chain	protein coding	This gene encodes one of the three alpha chains of type VI collagen, a beaded filament collagen found in most connective tissues. The product of this gene contains several domains similar to von Willebrand Factor type A domains. These domains have been shown to bind extracellular matrix proteins, an interaction that explains the importance of this collagen in organizing matrix components. Mutations in this gene are associated with Bethlem myopathy and Ullrich scleroatonic muscular dystrophy. Three transcript variants have been identified for this gene. [provided by RefSeq, Jul 2008]
IGFBP4	3487	BP-4; IBP4; IGFBP-4; HT29-IGFBP	insulin like growth factor binding protein 4	protein coding	This gene is a member of the insulin-like growth factor binding protein (IGFBP) family and encodes a protein with an IGFBP domain and a thyroglobulin type-I domain. The protein

					binds both insulin-like growth factors (IGFs) I and II and circulates in the plasma in both glycosylated and non-glycosylated forms. Binding of this protein prolongs the half-life of the IGFs and alters their interaction with cell surface receptors. [provided by RefSeq, Jul 2008]
INSC	387755	-	INSC spindle orientation adaptor protein	protein coding	In Drosophila, neuroblasts divide asymmetrically into another neuroblast at the apical side and a smaller ganglion mother cell on the basal side. Cell polarization is precisely regulated by 2 apically localized multiprotein signaling complexes that are tethered by Inscuteable, which regulates their apical localization (Izaki et al., 2006 [PubMed 16458856]).[supplied by OMIM, Mar 2008]

Table S4. Genes composing the 25-gene osteoclast-specific gene signature. Gene symbol, gene ID, synonyms, full name(s), information on the gene type, and a short description from NCBI Gene are provided.

Symbol	Gene ID	Synonym	Name	Gene type	Description/Summary
CHIT1	1118	CHI3; CHIT; CHITD	Chitinase 1	Protein coding	Chitotriosidase is secreted by activated human macrophages and is markedly elevated in plasma of Gaucher disease patients. The expression of chitotriosidase occurs only at a late stage of differentiation of monocytes to activated macrophages in culture. Human macrophages can synthesize a functional chitotriosidase, a highly conserved enzyme with a strongly regulated expression. This enzyme may play a role in the degradation of chitin-containing pathogens. Several alternatively spliced transcript variants have been described for this gene. [provided by RefSeq, Jan 2012]
DCSTAMP	81501	FIND; TM7SF4; hDC-STAMP	dendrocyte expressed seven transmembrane protein	Protein coding	This gene encodes a seven-pass transmembrane protein that is primarily expressed in dendritic cells. The encoded protein is involved in a range of immunological functions carried out by dendritic cells. This protein plays a role in osteoclastogenesis and myeloid differentiation. Alternate splicing results in multiple transcript variants. [provided by RefSeq, Mar 2012]
AC004988.1	-	-	-	-	-
C11orf45	219833	KCNJ5-AS1	KCNJ5 antisense RNA 1	ncRNA	Predicted to be located in extracellular region. [provided by Alliance of

					Genome Resources, Apr 2022]
SUCNR1	56670	GPR91	succinate receptor 1	protein coding	This gene encodes a G-protein-coupled receptor for succinate, an intermediate molecule of the citric acid cycle. It is involved in the promotion of hematopoietic progenitor cell development, and it has a potential role in renovascular hypertension which has known correlations to renal failure, diabetes and atherosclerosis. [provided by RefSeq, Oct 2009]
ADAMDEC1	27299	M12.219	ADAM like decysin 1	protein coding	This encoded protein is thought to be a secreted protein belonging to the disintegrin metalloproteinase family. Its expression is upregulated during dendritic cells maturation. This protein may play an important role in dendritic cell function and their interactions with germinal center T cells. [provided by RefSeq, Jul 2008]
GAL	51083	ETL8; GALN; GLNN; GMAP; GAL-GMAP	galanin and GMAP prepropeptide	protein coding	This gene encodes a neuroendocrine peptide that is widely expressed in the central and peripheral nervous systems and also the gastrointestinal tract, pancreas, adrenal gland and urogenital tract. The encoded protein is a precursor that is proteolytically processed to generate two mature peptides: galanin and galanin message-associated peptide (GMAP). Galanin has diverse physiological functions including nociception, feeding and energy homeostasis, osmotic regulation and water balance. GMAP has been demonstrated to possess antifungal activity

					and hypothesized to be part of the innate immune system. [provided by RefSeq, Jul 2015]
SLC28A3	64078	CNT3	solute carrier family 28 member 3	protein coding	Nucleoside transporters, such as SLC28A3, regulate multiple cellular processes, including neurotransmission, vascular tone, adenosine concentration in the vicinity of cell surface receptors, and transport and metabolism of nucleoside drugs. SLC28A3 shows broad specificity for pyrimidine and purine nucleosides (Ritzel et al., 2001 [PubMed 11032837]).[supplied by OMIM, Mar 2008]
PLA2G7	7941	PAFAD; PAFAH; LP-PLA2; LDL-PLA2	phospholipase A2 group VII	protein coding	The protein encoded by this gene is a secreted enzyme that catalyzes the degradation of platelet-activating factor to biologically inactive products. Defects in this gene are a cause of platelet-activating factor acetylhydrolase deficiency. Two transcript variants encoding the same protein have been found for this gene.[provided by RefSeq, Dec 2009]
TREM2	54209	PLOSL2; TREM-2; Trem2a; Trem2b; Trem2c	triggering receptor expressed on myeloid cells 2	protein coding	This gene encodes a membrane protein that forms a receptor signaling complex with the TYRO protein tyrosine kinase binding protein. The encoded protein functions in immune response and may be involved in chronic inflammation by triggering the production of constitutive inflammatory cytokines. Defects in this gene are a cause of polycystic lipomembranous osteodysplasia with sclerosing

					leukoencephalopathy (PLOSL). Alternative splicing results in multiple transcript variants encoding different isoforms. [provided by RefSeq, Nov 2012]
AL121933	-	-	-	-	-
ACP5	54	HPAP; TRAP; TRAcP; TRACP5a; TRACP5b; TrATPase	acid phosphatase 5, tartrate resistant	protein coding	This gene encodes an iron containing glycoprotein which catalyzes the conversion of orthophosphoric monoester to alcohol and orthophosphate. It is the most basic of the acid phosphatases and is the only form not inhibited by L(+)-tartrate. [provided by RefSeq, Aug 2008]
SDCBPP2	100129960	-	syndecan binding protein pseudogene 2	pseudo	-
CYP27B1	1594	VDR; CP2B; CYP1; PDDR; VDD1; VDDR; VDDRI; CYP27B; P450c1; CYP1alpha	cytochrome P450 family 27 subfamily B member 1	protein coding	This gene encodes a member of the cytochrome P450 superfamily of enzymes. The cytochrome P450 proteins are monooxygenases which catalyze many reactions involved in drug metabolism and synthesis of cholesterol, steroids and other lipids. The protein encoded by this gene localizes to the inner mitochondrial membrane where it hydroxylates 25-hydroxyvitamin D3 at the 1alpha position. This reaction synthesizes 1alpha,25-dihydroxyvitamin D3, the active form of vitamin D3, which binds to the vitamin D receptor and regulates calcium metabolism. Thus this enzyme regulates the level of biologically active vitamin D and plays an important role in calcium

					homeostasis. Mutations in this gene can result in vitamin D-dependent rickets type I. [provided by RefSeq, Jul 2008]
MMP7	4316	MMP-7; MPSL1; PUMP-1	matrix metallopeptidase 7	protein coding	This gene encodes a member of the peptidase M10 family of matrix metalloproteinases (MMPs). Proteins in this family are involved in the breakdown of extracellular matrix in normal physiological processes, such as embryonic development, reproduction, and tissue remodeling, as well as in disease processes, such as arthritis and metastasis. The encoded preproprotein is proteolytically processed to generate the mature protease. This secreted protease breaks down proteoglycans, fibronectin, elastin and casein and differs from most MMP family members in that it lacks a conserved C-terminal hemopexin domain. The enzyme is involved in wound healing, and studies in mice suggest that it regulates the activity of defensins in intestinal mucosa. The gene is part of a cluster of MMP genes on chromosome 11. This gene exhibits elevated expression levels in multiple human cancers. [provided by RefSeq, Jan 2016]
NCAPH	23397	CAPH; BRRN1; CAP-H; MCPH23	non-SMC condensin I complex subunit H	protein coding	This gene encodes a member of the barr gene family and a regulatory subunit of the condensin complex. This complex is required for the conversion of interphase chromatin

					into condensed chromosomes. The protein encoded by this gene is associated with mitotic chromosomes, except during the early phase of chromosome condensation. During interphase, the protein has a distinct punctate nucleolar localization. Alternatively spliced transcript variants encoding different proteins have been described. [provided by RefSeq, Jul 2013]
CCL22	6367	MDC; ABCD-1; SCYA22; STCP-1; DC/B-CK; A-152E5.1	C-C motif chemokine ligand 22	protein coding	This antimicrobial gene is one of several Cys-Cys (CC) cytokine genes clustered on the q arm of chromosome 16. Cytokines are a family of secreted proteins involved in immunoregulatory and inflammatory processes. The CC cytokines are proteins characterized by two adjacent cysteines. The cytokine encoded by this gene displays chemotactic activity for monocytes, dendritic cells, natural killer cells and for chronically activated T lymphocytes. It also displays a mild activity for primary activated T lymphocytes and has no chemoattractant activity for neutrophils, eosinophils and resting T lymphocytes. The product of this gene binds to chemokine receptor CCR4. This chemokine may play a role in the trafficking of activated T lymphocytes to inflammatory sites and other aspects of activated T lymphocyte physiology. [provided by RefSeq, Sep 2014]

SLC38A6	145389	NAT-1; SNAT6	solute carrier family 38 member 6	protein coding	Predicted to enable L-glutamine transmembrane transporter activity. Predicted to be involved in amino acid transmembrane transport and glutamine transport. Predicted to be located in plasma membrane. Predicted to be integral component of plasma membrane. [provided by Alliance of Genome Resources, Apr 2022]
ATP6V0D2	245972	VMA6; ATP6D2	ATPase H+ transporting V0 subunit d2	protein coding	Predicted to enable proton transmembrane transporter activity. Predicted to be involved in vacuolar acidification and vacuolar transport. Located in apical plasma membrane. Part of vacuolar proton-transporting V-type ATPase complex. [provided by Alliance of Genome Resources, Apr 2022]
SULT1C2	6819	ST1C1; ST1C2; SULT1C1; humSULTC2	sulfotransferase family 1C member 2	protein coding	Sulfotransferase enzymes catalyze the sulfate conjugation of many hormones, neurotransmitters, drugs, and xenobiotic compounds. These cytosolic enzymes are different in their tissue distributions and substrate specificities. The gene structure (number and length of exons) is similar among family members. This gene encodes a protein that belongs to the SULT1 subfamily, responsible for transferring a sulfo moiety from PAPS to phenol-containing compounds. Two alternatively spliced transcript variants encoding different isoforms have been described for this gene.

					[provided by RefSeq, Jul 2008]
MMP12	4321	ME; HME; MME; MMP-12	matrix metallopeptidase 12	protein coding	This gene encodes a member of the peptidase M10 family of matrix metalloproteinases (MMPs). Proteins in this family are involved in the breakdown of extracellular matrix in normal physiological processes, such as embryonic development, reproduction, and tissue remodeling, as well as in disease processes, such as arthritis and metastasis. The encoded preproprotein is proteolytically processed to generate the mature protease. This protease degrades soluble and insoluble elastin. This gene may play a role in aneurysm formation and mutations in this gene are associated with lung function and chronic obstructive pulmonary disease (COPD). This gene is part of a cluster of MMP genes on chromosome 11. [provided by RefSeq, Jan 2016]
HTRA4	203100	-	HtrA serine peptidase 4	protein coding	This gene encodes a member of the HtrA family of proteases. The encoded protein contains a putative signal peptide, an insulin growth factor binding domain, a Kazal protease inhibitor domain, a conserved trypsin domain and a PDZ domain. Based on studies on other related family members, this enzyme may function as a secreted oligomeric chaperone protease to degrade misfolded secretory proteins. Other human HtrA proteins have been implicated in arthritis, tumor suppression, unfolded stress response,

					apoptosis, and aging. [provided by RefSeq, Oct 2008]
HK3	3101	HXK3; HKIII	hexokinase 3	protein coding	Hexokinases phosphorylate glucose to produce glucose-6-phosphate, the first step in most glucose metabolism pathways. This gene encodes hexokinase 3. Similar to hexokinases 1 and 2, this allosteric enzyme is inhibited by its product glucose-6-phosphate. [provided by RefSeq, Apr 2009]
CHI3L1	1116	GP39; ASRT7; GP-39; YK-40; YKL40; CGP-39; YKL-40; YYL-40; HC-gp39; HCGP-3P; hCGP-39	chitinase 3 like 1	protein coding	Chitinases catalyze the hydrolysis of chitin, which is an abundant glycopolymer found in insect exoskeletons and fungal cell walls. The glycoside hydrolase 18 family of chitinases includes eight human family members. This gene encodes a glycoprotein member of the glycosyl hydrolase 18 family. The protein lacks chitinase activity and is secreted by activated macrophages, chondrocytes, neutrophils and synovial cells. The protein is thought to play a role in the process of inflammation and tissue remodeling. [provided by RefSeq, Sep 2009]
MMP9	4318	GELB; CLG4B; MMP-9; MANDP2	matrix metallopeptidase 9	protein coding	Proteins of the matrix metalloproteinase (MMP) family are involved in the breakdown of extracellular matrix in normal physiological processes, such as embryonic development, reproduction, and tissue remodeling, as well as in disease processes, such as arthritis and metastasis. Most MMP's are secreted as inactive proproteins which are activated when cleaved by extracellular

					proteinases. The enzyme encoded by this gene degrades type IV and V collagens. Studies in rhesus monkeys suggest that the enzyme is involved in IL-8-induced mobilization of hematopoietic progenitor cells from bone marrow, and murine studies suggest a role in tumor-associated tissue remodeling. [provided by RefSeq, Jul 2008]

Table S5: Key information on GSE data sets used in this study. The information was derived from the corresponding original publication, indicated by the DOI number, and the Gene Expression Omnibus (GEO) repository.

GSE number	Cell type	Stimulus	Time points	Number of biological replicates	Cell culture	Publication (DOI)
GSE12264	osteoblasts	untreated	-	4	cell culture in differentiation and mineralization medium without or with β-glycerophosphate	10.1089/ten.TEC.2009.0405
	osteoblasts	10 mM β-glycerophosphate	early time point 24 h	4		10.1089/ten.TEC.2009.0405
	osteoblasts	10 mM β-glycerophosphate	intermediate time point 7 d	4		10.1089/ten.TEC.2009.0405
	osteoblasts	10 mM β-glycerophosphate	late time point 10-14 d	2		10.1089/ten.TEC.2009.0405
GSE63009	osteoclast	mock untreated	-	3	cell culture without or with alendronate or risedronate	10.1073/pnas.1421410111
	osteoclast	100 nM alendronate	8 d	3		10.1073/pnas.1421410111
	osteoclast	100 nM risedronate	8 d	3		10.1073/pnas.1421410111
GSE10311	osteoblasts	untreated	-	3	cell culture without or with BMP-2 or dexamethasone	10.1152/physiolgenomics.00028.2008
	osteoblasts	mock treated	2 h	3		10.1152/physiolgenomics.00028.2008
	osteoblasts	mock treated	24 h	3		10.1152/physiolgenomics.00028.2008
	osteoblasts	10^{-4} mg/ml of bone morphogenic protein (BMP)-2	2 h	3		10.1152/physiolgenomics.00028.2008
	osteoblasts	10^{-4} mg/ml of bone morphogenic protein (BMP)-2	24 h	3		10.1152/physiolgenomics.00028.2008
	osteoblasts	10^{-7} M of dexamethasone	2 h	3		10.1152/physiolgenomics.00028.2008
	osteoblasts	10^{-7} M of dexamethasone	24 h	3		10.1152/physiolgenomics.00028.2008
